# Student Research and Publication: Strategic Planning for Inclusion Using a Systems Mapping Approach

**DOI:** 10.3389/fpsyg.2019.00006

**Published:** 2019-01-22

**Authors:** Emily Chan

**Affiliations:** Department of Psychology, Colorado College, Colorado Springs, CO, United States

**Keywords:** student research, climate, strategic planning, diversity, inclusion, publication

Students who are first-generation, from historically underrepresented groups, or from lower income backgrounds tend to be underrepresented in the participation of high impact educational practices (Stableton and Soria, 2012). In particular and relevant to the current volume, they publish comparatively less as student co-authors (Grineski et al., 2018). As faculty, we should examine where the leaks are along the pipeline of student research, from recruitment to publication. In addition to leading department level programming, faculty can be powerful advocates for institution level action that synergizes individual and departmental practices (Morales et al., 2017). This article will suggest strategic planning steps as well as actions for implementation that create a positive and inclusive climate along the entire undergraduate research pipeline from entry into research experiences to publication.

## Step 1: What is the Climate? A Multi-systems Mapping Exercise

Faculty create and adopt impactful practices to foster undergraduate research success at the classroom and departmental level [e.g., incorporation of research into courses (LoSchiavo, 2018, this volume; Sharen et al., 2017) and integrated curriculum design (McKelvie and Standing, 2018), this volume]. However, to ensure that the impact is inclusive, we should consider how individual practices fit into the institution's climate.

The Systems View of School Climate (Rudasill et al., 2018) provides a practical framework for this mapping exercise. It posits that students' perception of climate is shaped at multiple levels. The *microsystem* is the most immediate context and is where faculty-led actions that directly impact student experiences typically reside. For example, this includes departmental policies and practices that encourage or restrict student research, such as mentoring programs, expectations and requirements, and peer learning communities. Wayment and Dickson (2008) describe a successful case of a departmental effort to increase student research participation by identifying existing microsystem barriers (student awareness, student access, curricular timing, publicity, and faculty incentives) and implementing targeted changes to remove the barriers (advertisement, application procedures, assessment and communication, newsletter, and faculty teaching load reassignment).

Nested within microsystems are *nanosystems* (e.g., identity-based peer groups, interest, or affinity within the major; tracks of study within the major) which affect students' identities and sense of belonging and in turn impact students' aspirations and attainment in academic and career outcomes such as engaging in research (Fisher et al., 2017). At the emergent level above microsystems, multiple microsystems such as different academic departments, administrative/student life offices, and students' family environments interact to create the *mesosystem*. For example, do departments share mentoring practices for student researchers or are students hearing different messages from different departments? Do students get advice aboutresearch vs. internship opportunities that are at odds? Are financial and financial aid processes supportive for student research assistants or those who receive research grants? Do families understand the value of student research opportunities? Messages and practices are sometimes mutually reinforcing and encourage student research engagement, but other times the expectations and values from microsystems can conflict. Mapping a systems view is therefore crucial for sustained progress in encouraging undergraduate research that can lead to publication, because while practices adopted within the department could improve the climate at the microsystem level, they could end up with limited impact if other microsystems, nanosystems, and mesosystems are at odds.

Faculty interested in understanding their institutional climate for student research could consider this mapping exercise to identify the multiple layers of systems in their own department and institution. At this stage, critical questions include:

- What are the microsystems involved? E.g., your own department, other science and social science departments, student service offices, administrative offices.
- What are the nanosystems within your department? How do they extend beyond your department and cut across departments?
- Where do the microsystems interact and what are the alignments and conflicts?
- At each level, can you begin to identify positive practices and potential obstacles?

## Step 2: Climate and Engagement Mapping–Dashboard and Gap Analysis

In order to effectively prioritize initiatives, faculty should also collaborate with institutional research offices to map the participation rates and diversity along the research pipeline of recruitment, retention, and advancement. By comparing the profiles at different points in the pipeline to the institution's demographics as a baseline, resources can be targeted at the leaky parts of the pipeline. Each department will need to identify the unique key points in its departmental and institutional pipeline, but some common metrics for such a dashboard could include the total number and the rates for student subgroups of interest (e.g., gender, race, ethnicity, international, first-generation status, Pell-eligibility):

- Retention rate within the major (expression of interest vs. graduating with the major) in psychology or in the sciences
- Participation in any kind of research (course-based and independent research)
- Participation as paid student assistants, or credit-gaining research assistant experiences
- Participation in summer research
- Receiving institutional funds for research expenses
- Receiving departmental or institutional awards for academic excellence
- Presentation as authors or co-authors at regional and national conferences
- Co-authorship with faculty.

Some of the data from this analysis could be posted as public information as a departmental or divisional dashboard to create a climate of transparency, collaboration, and accountability (e.g., https://www.coloradocollege.edu/offices/dean/students/research-opportunities/score-facts). These findings, along with departmental/institutional data on student engagement with High Impact Practices (Kuh, 2008), qualitative data from student focused groups, and campus climate surveys will give insights into the nano, micro, and mesosphere factors affecting the student research pipeline.

## Step 3: Strategic Planning: Early, Mid, and Advanced Stages of the Pipeline

Equipped with the qualitative and quantitative understanding of the climate and practices along the research pipeline, faculty can then strategize based on the nature of the gaps and patterns within and across departments.

### Early Pipeline

How does the department recruit students into the discipline and its research experiences? Entry experiences across different departments and programs affect the mesosystem climate by shaping student expectations and identity (Oyserman et al., 1995). Undergraduate students who participate in research tend to enter in their later years because research is typically structured as capstone experiences (Kenny et al., 2001). However, efforts to broaden and diversify the pipeline in the senior year may be too late. At our college, after quantitative study identified the gaps in undergraduate research, follow-up student surveys and focus groups suggested that first generation and minority students are often uncertain about who to and how to ask for research experiences, and some worry that they lack experience to start research. As a result, we implemented a pre-major advising program with and mentoring to help students navigate “how to get started” (https://www.coloradocollege.edu/offices/dean/students/research-opportunities/getting-started/).

We also began a science research apprenticeship program for first year students that provided paid student-employment positions for work-study eligible students. It is important that these are paid, not volunteer, positions because competing job responsibilities is a major academic obstacle for first-generation students (Stableton and Soria, 2012). In our program, faculty from psychology and other science departments were recruited to offer novice-level research assistant positions that were centrally funded. The postings and marketing for these apprenticeships were centralized, and each department did its own review, interviews, and selection. Students then began work in labs in mid-October under faculty mentorship while participating in professional development opportunities as a cohort. The initial cohort revealed the challenges of incorporating first years in research because of the variability in each student's knowledge in the discipline, as well as the availability of novice-level work that a student can perform in each research field. In addition to directly engaging the cohort of first year research students, their early involvement and positive experience in the science community should positively impact the nanosystem climate for other first-generation and historically underrepresented students.

### Mid Pipeline

This part of the pipeline focuses on retention—how to foster sustained student engagement to produce work of publishable quality. Having a summer undergraduate research program is essential for long-term and focused research experiences (Rowlett et al., 2012) and some institutions further fund undergraduates to present their research at national conferences. However, while conference attendance is effective in motivating students to sustain their research after the summer, it is resource intensive and impacts only a small group of students. A scalable and economical mesosystem solution is to create a prominent campus symposium on undergraduate research co-hosted by administrative offices such as the academic affairs division, alumni office, advancement division, and career center. Such a symposium should ideally be run as a central part of an existing campus event, such as a fall semester Family Weekend or Homecoming Weekend, to maximize its impact on the mesosystem climate. At our college, we developed an Undergraduate Research and Internships Symposium as a major event for our Family Weekend. A few students deliver high quality oral presentations, followed by poster sessions showcasing students who received institutional funding for summer research or internships. The event has been well-attended by student peers, faculty, staff, and families and friends of the student researchers.

A symposium that is well-integrated at the mesosystem can align initiatives from multiple microsystems by creating synergies across academic departments and administrative offices. It also positively affects the nanosystems—The expectation to present their summer research early in the fall semester formed a learning community of student researchers that shared the experience of struggle and perseverance through challenges (deadlines, learning how to make posters, practicing public speaking); the event created a space where the voices and achievements of students from historically underrepresented groups can be recognized (especially when earlier pipeline issues are addressed and presenters represent campus student demographics); students not yet involved with research can encounter peer role models within and across departments; families can witness how student research and internships work side by side to promote postgraduate success.

### End of Pipeline

Toward the end of the pipeline, what can create accountability and community to encourage publication? Returning to interactions between nano and microsystems, Grineski et al. (2018) points out that cultural factors associated with socioeconomic status complicate the way well-intentioned faculty-level actions might still fall short in terms of equitable and inclusive student publication rates—first-generation students were significantly less likely to publish, even after accounting for factors such as confidence, duration of research, mentoring, and major.

Research with graduate students found that writing groups and programs lead to increased publications (Cuthbert and Spark, 2008; Cargill and Smernik, 2016). This practice could be adapted for undergraduates by forming scholarly writing groups that are only for undergraduates, or by introducing undergraduates into existing writing communities of graduate students and faculty. The writing program should include community accountability (e.g., daily writing goals) as well as skill-building components that help undergraduates become better writers and editors of their writing. Opportunities for undergraduates to earn academic credit for this intensive writing and rewriting for publication will provide additional accountability. For institutions with a culture of student research grants, explicitly communicating grant availability for funding publication fees will further highlight the cross-system institutional support for student research publication.

## Conclusion

As faculty design and implement new ideas to foster student research, multi-system awareness will help faculty attend to the overall participation, inclusion, and effectiveness. The nature of student research is that only a small fraction will end up in publications, and therefore it is imperative to monitor for inclusion along all parts of the pipeline and adopt practices to ensure that no groups suffer disproportionate attrition in the research experience, and the opportunity to publish is attainable in an equitable way. With the changing demographics of the college-aged population in the next decade, strategic planning that accounts for inclusion and multisystem dynamics will create sustainable long-term success for students.

## Author Contributions

The author confirms being the sole contributor of this work and has approved it for publication.

### Conflict of Interest Statement

The author declares that the research was conducted in the absence of any commercial or financial relationships that could be construed as a potential conflict of interest.
